# The Impact of Nutrition on the COVID-19 Pandemic and the Impact of the COVID-19 Pandemic on Nutrition

**DOI:** 10.3390/nu13061752

**Published:** 2021-05-21

**Authors:** Delfin Rodriguez-Leyva, Grant N. Pierce

**Affiliations:** 1 Department of Physiology and Pathophysiology, Rady Faculty of Health Sciences, University of Manitoba, Winnipeg, MB R3E 0W3, Canada; drodriguez@sbrc.ca; 2 Department of Internal Medicine, Rady Faculty of Health Sciences, University of Manitoba, Winnipeg, MB R3E 0W3, Canada; 3 Institute of Cardiovascular Sciences, Albrechtsen Research Centre, St. Boniface Hospital, 351 Tache Avenue, Winnipeg, MB R2H 2A6, Canada; 4 Canadian Centre for Agri-Food Research in Health and Medicine (CCARM), Albrechtsen Research Centre, St. Boniface Hospital, 351 Tache Avenue, Winnipeg, MB R2H 2A6, Canada

**Keywords:** nutrition, COVID-19, pandemic, review, food

## Abstract

The coronavirus disease 2019 (COVID-19) pandemic has had significant morbidity, mortality, social and financial implications for the global population. Despite this knowledge, we still know very little about how COVID-19 infection affects quality of life resulting from changes in nutritional behaviour and, conversely, how nutrition could modulate the epidemiology of COVID-19. In addition, the social isolation most have experienced due to the regulations imposed by governments during the COVID-19 pandemic may have also had effects on our nutritional behaviour. It is possible that nutritional interventions may have effects on the incidence of COVID-19 infection and mortality rates. The purpose of this review is to evaluate the current status of research on the topic of nutrition as it relates to the COVID-19 pandemic.

## 1. Introduction

As identified in the Global Burden of Disease Study 2016, dietary habits represent the second most important risk factor for determining mortality and disability-adjusted life-years in the world [1]. Any disruption in nutrition, therefore, will have a significant immediate and long-term impact on health. Conversely, improvements in nutritional behaviour can have a beneficial therapeutic effect in populations both with and without clinical symptoms.

At the termination of 2019 and throughout 2020, the spread of the coronavirus disease 2019 (COVID-19) virus from first identification in China across the world has had a major health and financial impact that the world has not seen for at least a century. This is expected to continue well into 2021 and probably 2022 as well. The potential impact of COVID-19 on nutritional habits and, inversely, the impact of nutrition on the epidemiology of the COVID-19 pandemic is worthy of study considering the positive and negative associations of nutrition with morbidity and mortality. The purpose of this review is to evaluate nutritional research nutrition as it relates to the COVID-19 pandemic.

## 2. Material and Methods

A systematic review was performed. The literature search when it was relevant to COVID-19 was limited to the period of COVID-19 infection to date (2020 until February 2021). The literature search was conducted through electronic databases including PubMed, Google Scholar and Web of Science. All case reports, reviews and retrospective and prospective studies on the topic were reviewed. Keywords used to search the literature database were “COVID-19” or “COVID” or “H1N1” or “Ebola” or “influenza” and “nutrition”, or “food”, or “diet” or “food security”. Because research on the topic and actual data was limited, all available papers were reviewed without exclusion as long as they were reported in a peer reviewed journal in the English language. Government reports (i.e., Centers for Disease Control and Prevention or World Health Organization) on the topic were also reviewed as appropriate to the topic. Two independent researchers searched the databases to avoid risk of bias.

## 3. Predictions from Past Pandemics

Our past history with global pandemics would strongly suggest that nutritional status would have important implications for population health even decades after the current COVID-19 viral pandemic has been controlled. The 1918 flu pandemic had significant effects on markers of nutritional status assessed in individuals 75 years and older after being exposed to the virus in utero, during infancy and early childhood. Using knee height as a marker of nutritional status, a significant depression in growth was found which was exaggerated by increasing severity of the flu symptoms [2]. Women were more affected than men. The depressed growth was not minor. The Dutch population took 40 years to gain a similar magnitude of growth in non-pandemic times as was lost by those infected by the 1918 flu [2]. This marker of nutritional status may associate with the incidence of disease. Prenatal exposure to the 1918 flu virus was associated with an increased incidence (>20%) of ischemic heart disease much later in the century in people aged 60 to 82 years old [3]. This time, men were more strongly affected than women [3].

The 1918 influenza pandemic is not isolated when considering the impact of nutritional status on disease outbreaks. More recent pandemics including those from H1N1 influenza, Swine flu, and the Ebola and Nipah viruses have all been impacted by questionable food security practices including distribution and food availability concerns [4]. This is particularly true in African countries stricken with Ebola virus where food security was negatively impacted by the pandemic, and governments appeared unprepared to address the nutritional problems facing their populations and to guide them nutritionally during the pandemic [5,6,7,8]. The result was malnutrition, particularly in the children of these regions [9]. The capacity for a variety of nutraceuticals and nutritional supplements to confer protection from and survival after infection by H1N1 has been demonstrated in animal studies [10,11,12]. Because of this, sales of these products have increased but suspicions have arisen regarding their actual efficacy against H1N1 in humans [13,14]. In trials of patients with Ebola virus infection, multivitamin therapy was associated with lower mortality rates [15]. In view of these results following past pandemics, it is prudent to consider the possibility that the current COVID-19 virus may have affected today’s population in a similar manner. The distinct possibility that the COVID-19 pandemic in any way altered dietary habits, or if nutritional alterations may impact COVID-19 transmission, morbidity or mortality are valuable questions that will be discussed in this review.

## 4. Nutrition as a Strategy for Coronavirus Disease 2019 (COVID-19) Therapy

The potential for food products, as functional foods or nutraceutical extracts from foods, to alleviate or modify COVID-19 transmission, morbidity or mortality is an especially attractive hypothesis when vaccines are not available. In reviewing the literature, the topic is replete with articles hypothesizing a beneficial effect of a healthy diet in reducing the incidence of COVID-19 infection and depressing its clinical symptoms [16,17,18,19,20,21,22,23,24,25,26,27,28,29,30]. These articles review past association of a variety of nutritional factors that may influence infections in general and hypothesize that these same associations may modify COVID-19 viral morbidity and mortality. With solely circumstantial evidence to support the assertions, the factors that have been suggested by these authors to have a protective effect against COVID-19 infection include ketogenic diets, tea bioactives, zinc and other micronutrients, Chinese medicinal herbs, resveratrol, silvestrol, lycorine, garlic, flavonoids, fresh fruits and vegetables, nuts, unsaturated fats and many more natural compounds and nutraceuticals. Factors suggested to have a negative impact on COVID-19 outcomes include concentrated juices, sugared beverages, saturated fats, obesity, malnutrition, and cachexia among other influences. None of these studies directly tested the hypotheses.

However, several studies have generated experimental data in examinations of the nutritional status of COVID-19 infected patients. The prevalence of malnutrition was 42% in patients hospitalized with COVID-19 [31]. Strikingly similar results (39% of COVID-19 infected patients exhibited malnutrition) were obtained in another study [32]. Malnutrition was detected in 67% of COVID-19 infected patients admitted into the intensive care unit (ICU) [31]. Moderate malnutrition was observed in 24% of COVID-19 infected patients and 18% had evidence of severe malnutrition. However, no significant association was identified between nutritional status and either clinical signs of COVID-19 infection [31] or COVID-19 severity [32]. Non-ICU COVID-19 infected patients with inadequate energy and protein diets tended to be older in age and exhibited a higher mortality rate than those patients with adequate levels [33,34].

Trace element status and clinical outcomes were followed in a unique study of 100 healthy and 100 COVID-19 infected pregnant women [35]. In the first and third trimesters, serum zinc was lower but serum copper and magnesium was higher in infected pregnant women compared to control healthy pregnant women. The serum zinc:copper ratio was significantly decreased in the infected women. This ratio correlated with disease severity in COVID-19 patients. Not surprisingly, the serum zinc and the zinc/copper ratio was negatively correlated with inflammatory markers like C-reactive protein in COVID-19 infected patients [35].

Supplementation of the diet with vitamin D has received particular attention as a potential therapy for COVID-19 infection. The data to support this contention is largely circumstantial and without solid evidence to support it. Presumptions that the mechanisms of action of vitamin D with other viruses may be relevant to COVID-19 infection are untested in clinical trials. The avian influenza A H5N1 is one example [36]. Vitamin D deficiencies are evident in other infections of the respiratory tract [37,38,39] but it is unclear if this is a cause and effect relationship or an indirect association. Even if it is a direct relationship, it is unproven if this extends to COVID-19. The reader is referred to a comprehensive review of the potential for vitamin D supplementation to alter the immune system during infections elsewhere [30].

Goncalves and co-workers found a prevalence of low vitamin D levels in obese elderly, critically ill ICU patients infected with COVID-19 [40]. They concluded vitamin D deficiency in this susceptible population may be an important factor in infection by a coronavirus [40]. Vitamin D deficiencies were also found in COVID-19 infected patients admitted to a tertiary hospital in Italy [41]. Kohlmeier used a state-by-state Mendelian randomization analysis of COVID-19 related mortality rates in African Americans in the US and reported that the greater incidence of COVID-19 deaths in northern states supports the contention that vitamin D deficiency contributes to their higher risk [42]. However, there are so many confounding factors that would play a role in this association that the conclusion that “vitamin D adequacy denies the virus an easy foothold and thereby slows spreading of the contagion” is weak at best.

Paradoxical findings have also been reported. The prevalence of vitamin D insufficiency, moderate or severe deficiency was not correlated with COVID-19 clinical symptoms or outcomes [41]. Instead, when confounding co-factors were taken into consideration, a significant positive association was found between in hospital mortality and increasing vitamin D levels [43]. It is reasonable to conclude that further study of the relationship of vitamin D status and COVID-19 morbidity and mortality is required to clearly determine if it is beneficial in the prevention or treatment of specifically COVID-19 infections.

## 5. Food Security in the COVID-19 Pandemic

The discussion of the impact of nutrition on COVID-19 infection and mortality rates, as discussed previously in this manuscript is one valid approach to the issue but the opposite is also worthy of study. It is very possible that the pandemic has influenced the world’s food security. Maintaining the security of our food supply and having ready access to appropriate amounts of safe food is of paramount importance to all peoples. According to the US Department of Agriculture there are four categories of food security (Table 1). It is possible through the various lines of evidence obtained in several surveys and studies conducted throughout the world to begin to understand the impact that the pandemic has had on nutritional behaviour and food security globally.

Several studies have investigated food security as it relates to COVID-19. First and foremost, there is no evidence that viral transmission of COVID-19 has occurred via food packaging [45,46,47]. The virus remains viable under controlled laboratory conditions for 24–72 h on plastic, stainless steel and cardboard matrices. This offers the remote potential for transmission of the virus via food packaging. However, no evidence for such transmission exists to date. Food workers practice conventional hygienic methods like the regular use of personal protective equipment (masks, eye wear, disposable gloves), hand washing, disinfectants, maintaining physical distance, frequent viral testing and refraining from entering the workplace with any symptoms of respiratory illness. These practices are no different to what would be expected to occur in any workplace.

Similarly, there is no evidence that viral transmission of COVID-19 has occurred through foods [47]. Although the COVID-19 virus was suspected to be transmitted to humans from bats, the CDC has stated that the risk of COVID-19 transmission from animals to humans is considered low [48]. Coronaviruses can only multiply in live animals and humans. Food provides an inadequate environment for the virus to live. Similarly, the risk of contracting live COVID-19 virus from ingesting food products like cooked meats is extremely unlikely.

**Figure 1:**
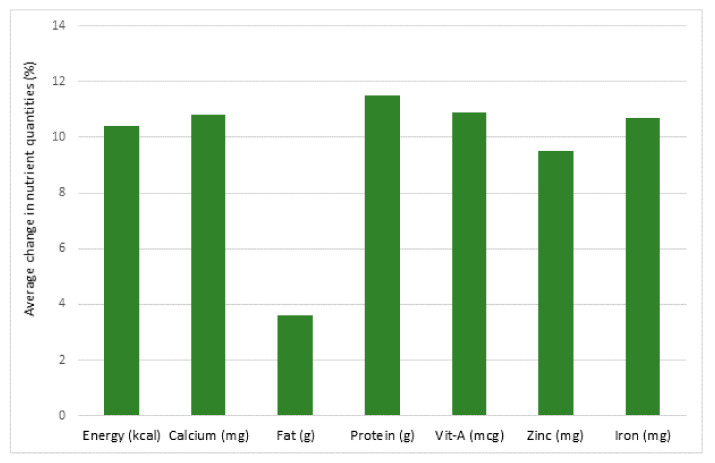
Average decrease in nutrient quantities (%) for school meals in Nepal between June 2019 (pre-coronavirus disease 2019 (COVID-19)) and May/June 2020 (post-COVID-19), due to food price inflation. (mg: milligram; mcg:microgram; g:gram; kcal:kilocalorie) [53].

Food security during a pandemic involves the stability of food availability in countries and communities as well. In Brazil, additional measures were implemented to increase food availability including a Basic Emergency Income program, a Food Acquisition Program and an emergency food donation program [49]. Nutritional inadequacies were reported in post-operative bariatric patients during the COVID-19 quarantine [50]. In Uganda, the pandemic caused delays to food distribution, poorer food quality, the use of mobile cash transfers to reduce corruption and provide economic assistance to families that were struggling with food insecurity [51]. In Jordan, surveys of more than 3000 citizens revealed 41% of the participants exhibited food security but 36% were moderately food insecure and 23% were severely insecure [52]. In Nepal, food prices increased and food quality and quality decreased significantly (Figure 1) during the pandemic [53]. Moderate food insecurity was associated with a monthly per capita income below the poverty line whereas a younger adult age (18–30 years old) and living in a rented house was significantly associated with severe food insecurity [52].

The nutritional response to the pandemic has been quite different in Europe and North America. In the US, the prevalence of food insecurity increased by one third from before to during the pandemic [54,55]. Even in the USA, disadvantaged communities have shown signs of severe food insecurity during the COVID-19 pandemic [56]. This appears to have been experienced across a wide age range of adults, from college students to the elderly [54].

## 6. Changes in Dietary Behaviour during the Pandemic and Their Consequences

In a Canadian study, the nutrient and caloric intake of university students were significantly reduced during the pandemic while alcohol intake increased significantly [57]. The frequency of consuming food groups across the board were decreased during the pandemic [57]. Low physical activity and increased sedentary activities were also observed. Conversely, in a Dutch study, overeating, primarily through snacking, was reported in 20–32% of respondents during the pandemic whereas 7–15% reported behaviour predisposed to undernutrition by skipping warm meals [58]. These changes in nutritional behaviour may be dependent upon the country examined. In Croatia, more than 4000 participants in a study revealed cooking frequency increased during the COVID-19 pandemic lockdown with an increased consumption of vegetables, legumes and seafood [59].

Social isolation during the pandemic-induced lockdown or quarantine resulted in more severe effects on nutritional status [58]. In a Belgian study, 10% of participants often or sometimes could not afford to eat a healthy diet during confinement [59]. Tendencies for fruit and vegetable consumption decreased and the consumption of soft drinks, sweets, bread and salty snacks increased [60,61,62]. Exercise in quarantine led to better nutritional choices which in turn had a beneficial effect on mental state as well [63]. However, it is important to emphasize that exercise frequency has been repeatedly reported to decrease during the pandemic [57,59] The poor nutritional behaviour and lack of exercise have resulted in predictable and unhealthy increases in weight gain during the pandemic [64,65]. These changes in dietary habits have important implications for the health impact of the pandemic. In countries across the world that had COVID-19 disease statistics collected by the WHO, infection and mortality rates due to COVID-19 were positively correlated with higher intake of fruits and sugar-sweetened beverages and negatively correlated with bean and legume consumption [54].

## 7. Conclusions

**Figure 2:**
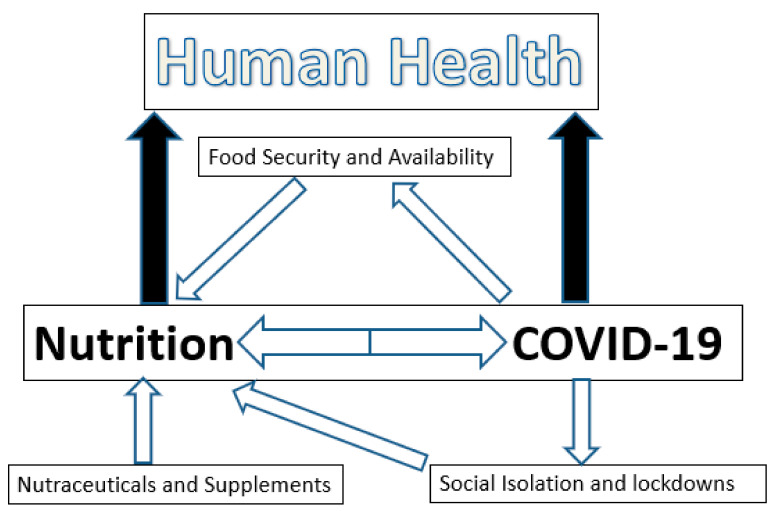
The interactions of nutrition and COVID-19 infection.

These and other studies taken together allow for the following conclusions (Figure 2) regarding the direct and indirect impact of the COVID-19 pandemic on food security:

1. The COVID-19 pandemic has caused changes in food supply. Special programs have been implemented in many countries to increase food availability and safety. Nonetheless, alterations in food availability due to the pandemic have occurred throughout the world. The severity depends upon the affluence and the economic status of the country. This has more serious implications particularly in third world countries as delays in food distribution, loss of food quality and quantity, impairments in food access and losses of income to purchase food have occurred. This has serious health implications acutely and, in view of the deleterious impacts that past pandemics have had on human growth and health, it is reasonable to predict that the current COVID-19 pandemic will induce nutritional deficiencies across the globe that will have a long-lasting negative impact on human health.
2. There is no evidence that the COVID-19 virus has been transmitted through foods or food packaging. This does not negate the importance of using appropriate precautionary measures in the food industry with, for example, the use of appropriate personal protective equipment, hand hygiene and disinfectants.
3. The nutritional status of a person can modulate infectious disease and the inflammatory processes associated with it positively or negatively by altering the immune system. Malnutrition in disadvantaged populations and in the elderly clearly leaves these populations more susceptible to COVID-19 infections and more severe clinical symptoms and outcomes. However, although infectivity rates and the severity of the clinical symptoms associated with a coronavirus infection may be modulated, it is highly unlikely that strong viral transmission can be fully prevented by following a healthy diet or supplementing the diet with nutraceuticals. The impact of vitamin D and zinc status in COVID-19 patients regarding viral transmission and its clinical symptoms remains unclear and requires further research.
4. COVID-19 has had a significant impact in some populations through altered eating behaviors. The impact of social isolation and lockdowns on eating behaviors during the COVID-19 pandemic should not be underestimated as it has already had acute effects and will likely produce long-term deleterious effects on population health as well. Poor nutritional choices sustained over extended periods of time will have increased plasma risk factors for cardiovascular disease, diabetes and cancer.

## Figures and Tables

**Table 1:** US Department of Agriculture categories of food security.

**High food security:** no reported indications of food-access problems or limitations;
**Marginal food security**: one or two reported indications—typically of anxiety over food sufficiency or shortage of food in the house. Little or no indication of changes in diets or food intake;
**Low food security**: reports of reduced quality, variety, or desirability of diet. Little or no indication of reduced food intake;
**Very low food security**: multiple indications of disrupted eating patterns and reduced food intake.

Table adapted from [44].

## Data Availability

Not applicable.
